# High Accuracy Mutation Detection in Leukemia on a Selected Panel of Cancer Genes

**DOI:** 10.1371/journal.pone.0038463

**Published:** 2012-06-04

**Authors:** Zeynep Kalender Atak, Kim De Keersmaecker, Valentina Gianfelici, Ellen Geerdens, Roel Vandepoel, Daphnie Pauwels, Michaël Porcu, Idoya Lahortiga, Vanessa Brys, Willy G. Dirks, Hilmar Quentmeier, Jacqueline Cloos, Harry Cuppens, Anne Uyttebroeck, Peter Vandenberghe, Jan Cools, Stein Aerts

**Affiliations:** 1 Center for Human Genetics, KU Leuven, Leuven, Belgium; 2 Center for the Biology of Disease, VIB, Leuven, Belgium; 3 Genomics Core Facility, University Hospitals Leuven, Leuven, Belgium; 4 Deutsche Sammlung von Mikroorganismen und Zellkulturen GmbH, Braunschweig, Germany; 5 Pediatric Oncology/Hematology and Hematology, VU Medical Center, Amsterdam, The Netherlands; 6 Pediatric Hemato-oncology, University Hospitals Leuven, Leuven, Belgium; Deutsches Krebsforschungszentrum, Germany

## Abstract

With the advent of whole-genome and whole-exome sequencing, high-quality catalogs of recurrently mutated cancer genes are becoming available for many cancer types. Increasing access to sequencing technology, including bench-top sequencers, provide the opportunity to re-sequence a limited set of cancer genes across a patient cohort with limited processing time. Here, we re-sequenced a set of cancer genes in T-cell acute lymphoblastic leukemia (T-ALL) using Nimblegen sequence capture coupled with Roche/454 technology. First, we investigated how a maximal sensitivity and specificity of mutation detection can be achieved through a benchmark study. We tested nine combinations of different mapping and variant-calling methods, varied the variant calling parameters, and compared the predicted mutations with a large independent validation set obtained by capillary re-sequencing. We found that the combination of two mapping algorithms, namely *BWA-SW* and *SSAHA2*, coupled with the variant calling algorithm *Atlas-SNP2* yields the highest sensitivity (95%) and the highest specificity (93%). Next, we applied this analysis pipeline to identify mutations in a set of 58 cancer genes, in a panel of 18 T-ALL cell lines and 15 T-ALL patient samples. We confirmed mutations in known T-ALL drivers, including PHF6, NF1, FBXW7, NOTCH1, KRAS, NRAS, PIK3CA, and PTEN. Interestingly, we also found mutations in several cancer genes that had not been linked to T-ALL before, including JAK3. Finally, we re-sequenced a small set of 39 candidate genes and identified recurrent mutations in TET1, SPRY3 and SPRY4. In conclusion, we established an optimized analysis pipeline for Roche/454 data that can be applied to accurately detect gene mutations in cancer, which led to the identification of several new candidate T-ALL driver mutations.

## Introduction

Next generation sequencing (NGS) technologies have significantly improved our sequencing capacity in the past five years. They are now widely used for research purposes and are starting to find their way into clinical applications. Although whole genome and whole exome sequencing approaches are successfully implemented for mapping the genomic landscapes of many human diseases, they are not routine strategies for detecting molecular aberrations due to high costs, and long turnover times (run and analysis times). Targeted re-sequencing, on the other hand, is appealing in a clinical setting, given the lower sequencing costs, shorter sequencing time and simpler data analysis. Moreover, as the discovery of novel cancer genes by whole-exome sequencing will gradually saturate and converge into a set of commonly mutated genes in a particular cancer, the identification of these mutations can yield important diagnostic and prognostic information.

Despite the requirement of several days for library preparation and target enrichment for all these platforms, the Roche/454 technology offers the advantages of short run times and data analysis time. In addition, the more restricted data output is also beneficial for turnaround time because fewer patient samples need to be collected to fill an entire sequencing run. Based on these advantages of the 454 platform for sequencing relatively small gene sets, we invested in optimizing bioinformatics pipelines for read mapping and variant calling of 454 reads, with the aim for applying this both for research as well as for clinical purposes. We focused on T cell acute lymphoblastic leukemia (T-ALL), an aggressive hematopoietic cancer caused by malignant transformation of developing T-cells [1]. A set of 97 genes was selected for targeted sequencing. The set consisted of 58 cancer genes [2] and 39 candidate genes including tyrosine kinase and phosphotase coding genes, chromatin modifiers, and several genes belonging to the families of known cancer driver genes such as TET1-TET3, or PIK3CB-PIK3CD-PIK3CG.

For accurate variant detection, we investigated several existing analysis pipelines and compared their performance. Although the companion software gsMapper is widely used in the analysis of 454 data [3], [4], [5], various alternative mapping and variant calling algorithms have been developed, such as BWA-SW [6] and SSAHA2 [7], BLAT [8] for mapping, and SAMTools [9], VarScan [10], and Atlas-SNP2 [11] for variant calling. Li et al [6] reviewed the long read aligners, and Shen et al [11] reviewed the variant callers, however, to our knowledge, no comparison has been performed on the combination of mapping and variant calling algorithms in the context of mutation discovery.

Here, we analyzed and compared nine different combinations of a mapping and variant calling algorithms and particularly investigated to what extent low coverage positions can be included in the variation calling process to increase the sensitivity of mutation detection. Next, we apply the optimized pipeline to identify mutations in a set of 58 cancer genes and 39 candidate genes, across 18 T-ALL cell lines and 15 T-ALL patient samples, and identify recurrent mutations in both known and novel drivers.

## Results

### Comparison of Mapping and Variation Calling Methods for Roche/454 Data

The Roche companion software *gsMapper* is mostly used for the analysis of Roche/454 data. This software first aligns the reads to the reference genome and then lists all positions that are different from the reference genome (variant calling). Although *gsMapper* performed well in several studies [3], [4], [5], we wanted to assess its performance on our data set and investigate whether we could achieve better precision and accuracy using alternative aligners and variant callers. We tested eight different combinations of a long read aligner (BWA-SW, SSAHA2, BLAT) and a variant caller (SAMTools, VarScan, Atlas-SNP2) and compared their performance with *gsMapper*.

Each pipeline was applied to the reads obtained from seven T-ALL cell lines and the performance of each pipeline was evaluated by Sanger re-sequencing of 210 candidate variants that were randomly taken from all predicted 8020 variants (containing both SNPs and mutations) from all pipelines. As a measure of the performance of each pipeline, we calculated the Matthews correlation coefficient (MCC), which is a measure of prediction accuracy that is calculated based on the number of successfully predicted true positives and true negatives found by Sanger sequencing (see Materials and Methods). When using default parameter settings (**Table S1**), the performance of the different pipelines was comparable, with an average MCC of 0.62, with no alternative pipeline performing better than gsMapper (MCC of 0.82) (**Table S1**).

In NGS studies, the presence of duplicate reads (caused by a PCR amplification step during library preparation) is a potential source of false positive single nucleotide variant (SNV) prediction [12]. Therefore, we added an additional step to remove duplicate reads using Picard, resulting in a 2–24% increase in MCC, depending on the pipeline, with an average MCC of 0.73 (**Table S1**). This showed that duplicate removal is an important step for obtaining correct variant calls.

**Figure 1:**
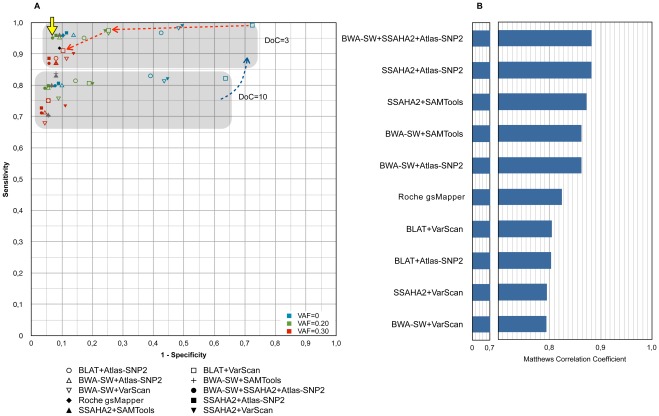
Performance comparison and parameter optimization. (A) Different pipelines show different sensitivity and specificity. Varying DoC and VAF thresholds in the variant calling process has an additional affect on the predictions in terms of sensitivity and specificity, respectively. Each pipeline is represented with a different symbol and the performance of each pipeline (in terms of sensitivity and specificity) is plotted under varying DoC and VAF thresholds. Note that the X-axis represents the false positive rate (1-specificity). In this ROC plot, the closer the point to the upper left point of the graph, the better the sensitivity and the specificity. Different colors of the symbols indicate the performance of the pipeline under changing VAF thresholds, and the two shaded boxes indicate the performance under changing DoC thresholds. The plot shows that (i) decreasing the DoC threshold increases the sensitivity of all pipelines as indicated with the blue dotted line; (ii) increasing the VAF threshold increases the specificity with a slight decrease in sensitivity as indicated (in the example of BLAT+VarScan pipeline) with the red dotted line; (iii) the BWA-SW+SSAHA2+Atlas-SNP2 pipeline has the best performance among all pipelines under DoC = 3 & VAF = 0.20 thresholds as indicated with the yellow arrow. The Roche pipeline is indicated with a black diamond shape since no parameter changes were performed on it, and SSAHA2+SAMTools and BWA-SW+SAMTools pipelines were colored grey since no VAF threshold changes were performed on them. (B) The Matthews correlation coefficient for each pipeline is shown for the most optimal performance of that pipeline (**Table S1**). It is interesting to note that the optimal performance of all the pipelines, except Roche gsMapper, was observed for a DoC threshold of 3.

Next, we further optimized the performance of each pipeline by varying the minimal required number of reads (depth of coverage, DoC) and the minimal required variant reads (variant allele frequency, VAF). Changes in DoC thresholds mainly affected the sensitivity, while varying VAF thresholds affected the predictions in terms of specificity (**Figure 1.A** **, Table S2**). All the pipelines reached their best performance with a DoC threshold of 3, and with a minimum VAF threshold of 0.20 (when applicable) (**Table S1-S2**). In a final effort to minimize false positive predictions, we combined the two best mapping algorithms in one pipeline, which further increased the sensitivity to 95% and the specificity to 93%. The reason for this increase in accuracy is that certain predicted variants that are caused by erroneous mapping **(Figure S1)** are now filtered out. Although this final pipeline (SSAHA2+ BWA-SW + Atlas-SNP2) performs better than *gsMapper* (91.2% sensitivity and 90.8% specificity), the difference is not large and *gsMapper* can be considered as a valid (and often easy to use) alternative (**Figure 1.B**).

### Widespread Mutations in Cancer Genes Across 18 T-ALL Cell Lines and 15 T-ALL Patient Samples

**Figure 2:**
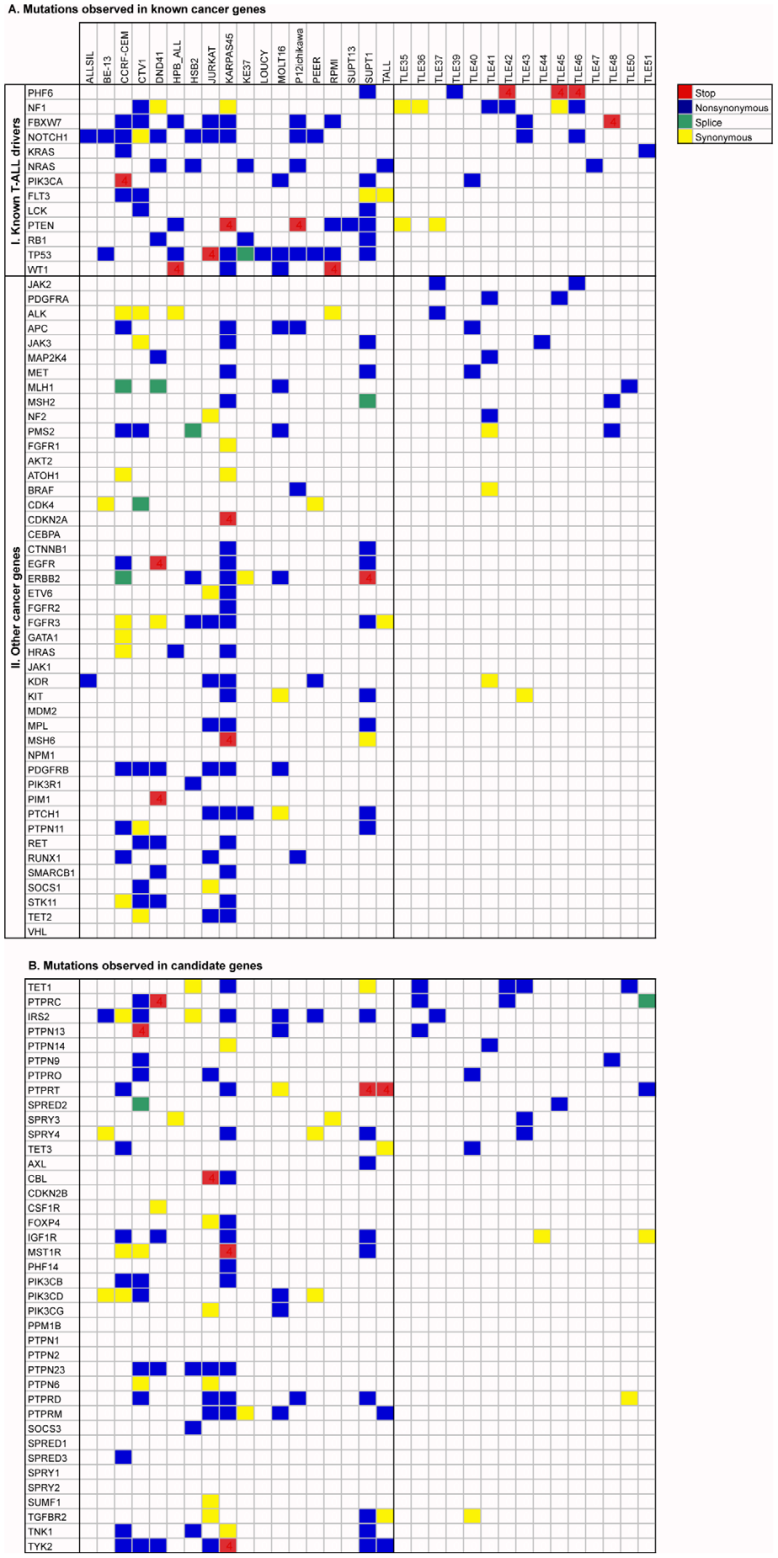
Mutations in the 97 genes. Coding mutations in known cancer genes (A) and candidate genes (B) are indicated with different color codes. Panel A is further subdivided into (I) genes that are known to be drivers in T-ALL, and (II) the genes that have recurrent somatic mutations in various human cancers. The cell lines are located to the left of the table, and the patient samples are located to the right. Genes are ranked according to the frequency of protein altering mutations in the patient samples.

We applied the optimized pipeline determined above, consisting of the SSAHA2+BWA-SW combination for read mapping, and Atlas-SNP2 for variation calling, to identify mutations in a panel of 58 “cancer genes” across 18 T-ALL cell lines and 15 primary T-ALL patient samples. This set of genes consists of 13 T-ALL drivers (**Figure 2.A.I**) and 45 other genes involved in a variety of cancers (**Figure 2.A.II**). All of these genes are present in the Census [2] database of cancer genes except for the recently discovered cancer genes ATOH1 and PHF6 [13], [14]. Since PHF6 mutations are involved in T-ALL we added PHF6 to our list of T-ALL drivers.

Sequence reads were mapped to the entire reference genome and those reads that map to the selected genes were retained. This resulted in 36% of reads that map to the target sequences on average, with an average coverage of 24.2X and 16.3X for cell lines and patient samples, respectively. Analysis of the sequence data revealed that exons with a very low coverage had a significantly higher GC-content compared to exons with higher coverage (p-value 2.2e-16), a finding consistent with a previously published study [15] (**Figure S2**). Of the 1565 exons targeted in this study, 18 exons had no coverage in the cell lines or in the patient samples (corresponding to 8710 bps); and 15 exons had no coverage in the patient samples only (corresponding to 5197 bps). On average, 94% and 86% of the targeted exons reached a mean coverage equal or above 3 for the cell lines and the patient samples, respectively.

Variation calling resulted in 836 distinct single nucleotide variants (SNVs) in known cancer genes across the 33 samples. Cell lines had significantly more SNVs in cancer genes than patient samples (p-value <0.001); on average 153 SNVs were detected per cell line and 117 per patient sample. 56% of the predicted SNVs were reported in dbSNP (http://www.ncbi.nlm.nih.gov/projects/SNP/) or in the 1000 Genomes project (http://www.1000genomes.org/) and were excluded from further analysis, while the remaining 368 SNVs **(Table S3)** affected 55 of the 58 sequenced cancer genes, primarily in the exons (58.4%) and in untranslated regions (23.9%). Furthermore, there were 8 SNVs affecting splice sites. Of the exonic SNVs, 14 result in the gain of a stop codon (called “stop gain” SNVs), 140 are non-synonymous and the remaining 61 are synonymous coding variations.

To validate the mutations found in cell lines, we compared our results with mutations determined by the Cancer Cell Line project [16], which contains eleven of our 18 cell lines. Of the 35 oncogenic point mutations found in the Cancer Cell line project (determined by capillary sequencing) in the genes that are included in our panel, 31 were recovered by the automated re-sequencing on Roche/454 using the SSAHA2+ BWA-SW + Atlas-SNP2 analysis pipeline, corresponding to a recovery rate of 88.5% **(Table S4)**. Note that gsMapper recovered 30 mutations out of 35, resulting in a recovery rate of 85.7%. The mutations that were missed by Roche/454 sequencing are either due to low coverage at those positions (in two of the four missed mutations, both in NOTCH1), or to low variant quality (one TP53 mutation), or to sequencing errors (one NOTCH1 mutation is covered by 10 reads, none of which contains the variant allele reported by the Cancer Cell line project). With regards to specificity, both pipelines performed well, for example on the FBXW7 gene for which we find a protein altering point mutation in exactly the same five cell lines as the Cancer Cell line project (out of the eleven common cell lines). In conclusion, the automated re-sequencing using Roche/454, with either the gsMapper pipeline or the SSAHA2+ BWA-SW + Atlas-SNP2 pipeline, is to a very large extent in agreement with mutations found by capillary sequencing.

Thirteen of the 58 cancer genes have been linked specifically to T-ALL, and we identified protein altering mutations in at least one of these genes in all cell lines and in 10 patient samples (**Figure 2.A.I**). Of the other 45 cancer genes, 36 genes were mutated (**Figure 2.A.II**), of which 25 were mutated in at least two samples (cell line or patient). The genes with most mutations in T-ALL cell lines are NOTCH1 (non-synonymous mutation in 9/18 cell lines), TP53 (10/18), FBXW7 (7/18), and NRAS (5/18). These also have mutations in patient samples, except TP53, suggesting that it may be easier to obtain cell lines from samples with TP53 mutation or that TP53 mutations are acquired during cell culture [17].

### Identification of Recurrent JAK3 Mutations in T-ALL

We next determined if mutations in cancer genes could be identified that were previously not linked to T-ALL. We found several such mutations in T-ALL cell lines (**Figure 2.A.II**), but their absence in the patient samples questions their relevance for the pathogenesis of T-ALL.

**Figure 3:**
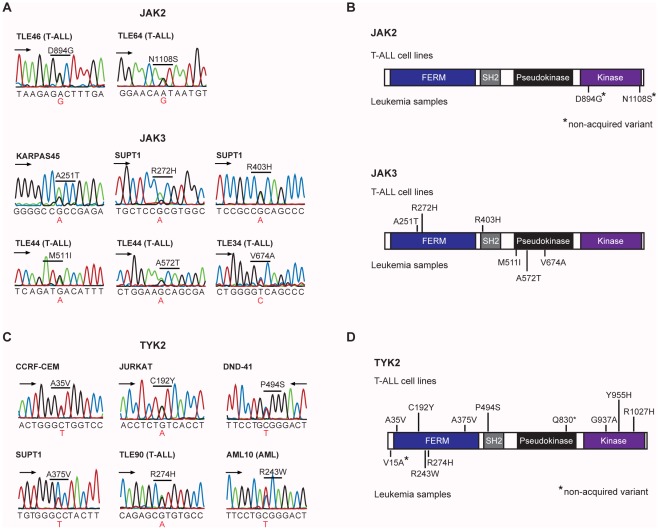
JAK kinase mutations. (A) Sanger sequencing chromatograms corresponding to confirmed JAK2/JAK3 variants. (B) Domain structure of JAK2 and JAK3 proteins with indication of novel detected variants. Non-somatic variants are indicated with an asterisk. (C) Sanger sequences showing examples of TYK2 variants detect in T-ALL cell lines or in leukemia patient samples. (D) Schematic representation of TYK2 protein structure with indication of all novel TYK2 variants detected in this study. Non-somatic variants are indicated with an asterisk.

We identified several mutations in JAK2 and JAK3 in both cell lines and patient samples. All JAK kinases, except TYK2 (see below), are known oncogenes in leukemia and activating mutations and translocations affecting JAK1, JAK2 and JAK3 were described in multiple, mainly myeloid, hematologic malignancies [18]. Until recently, JAK1 was the only JAK family member in which point mutations have been described in T-ALL [19]. However, in a recent article JAK3 gain-of-function mutations were described in T-ALL by Elliott et al. [20]. In our study, we have identified 3 non-synonymous coding mutations in 2 patients for JAK2 (patient TLE37 had two mutations) and 4 non-synonymous coding mutations in 1 patient and 2 cell lines (SUPT1 cell line had two mutations) for JAK3. **(Table S3)**. Sanger sequencing confirmed one JAK2 and all JAK3 variations **(Table S5,** **Figure 3.A-B** **)**. Complementary Sanger sequencing of all exons of the JAK2 and JAK3 genes in 31 additional T-ALL patients identified 1 additional JAK2 variant and 2 additional JAK3 variants **(Table S5,** **Figure 3.A-B** **)**. So, in total, we identified JAK2 mutations in 2 of 46 (4%) T-ALL samples and in 0 of 18 T-ALL cell lines and JAK3 mutations in 2 of 46 (4%) T-ALL samples and in 2 of 18 T-ALL cell lines **(Table S5,** **Figure 3.A-B** **)**. For JAK2, both mutations were also present in a corresponding remission sample, whereas all JAK3 patient mutations were somatically acquired. Interestingly, patient TLE44 showed 2 somatic mutations in JAK3, namely A572T and M511I, which were detected on the same allele (data not shown). Moreover, the M511I mutation has been detected before in AML and over-expression of this mutant transformed IL3 dependent 32D cells and induced T-ALL in mice [21]. Whereas the A572T mutation was not described before, JAK3 amino acid A572 was found mutated into a V (A572V mutation) in T-cell leukemia, T-cell lymphoma, and AML, and this A572V mutant transformed cytokine dependent hematopoietic cells and induced leukemia in mice [21], [22], [23], [24].

### Identification of New Oncogenes and Tumor Suppressor Genes in T-ALL

Searching for novel T-ALL driver genes can be performed by whole-exome sequencing or other genome-wide approaches. Nevertheless, the Roche/454 platform combined with sequence capture could be useful in a candidate gene approach. In our targeted re-sequencing approach, 39 genes were included that were not causally linked to cancer, but were selected as candidate oncogenes or tumor suppressor genes, because of their function (e.g., tyrosine kinases and tyrosine phosphatases) or because family members had been implicated in cancer (e.g., TYK2 for the JAK family, TET1 because TET2 is a known cancer gene). **Figure 2.B** indicates the exonic and splice site mutations observed in these genes and the genes were ranked according to the recurrence of protein altering variants across patient samples.

**Figure 4:**
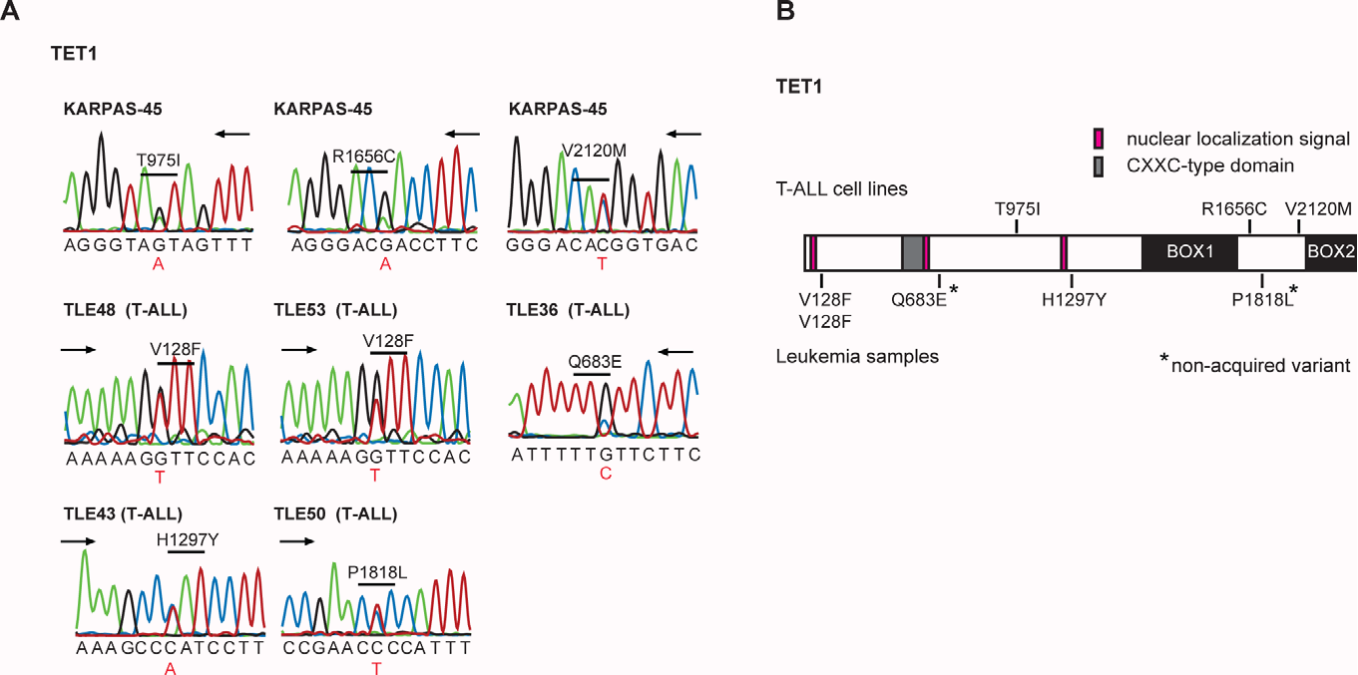
TET1 mutations in T-ALL. (A) Sanger sequencing chromatograms representing confimed TET1 variants. (B) Schematic representation of TET1 protein structure with indication of all novel TET1 variants detected in this study. Variants detected in cell lines are depicted above the TET1 protein, variants detected in leukemia patient samples are below the TET1 protein. Non-somatic variants are indicated with an asterisk.

Interestingly, 4 of the 15 sequenced patient samples contain a variation in TET1. The *TET* gene family (*TET1*, *TET2*, *TET3*) of epigenetic regulators is important for the hematology field because of the observation of *TET2* mutations in 10–25% patients with various myeloid hematologic diseases [25], [26], [27]. To better assess the mutation frequency of *TET1* in T-ALL, we performed supplemental Sanger sequencing of *TET1* in all cell lines and patient samples and in a panel of 22 additional T-ALL cases. Overall, this resulted in the identification of *TET1* variants in 5/37 (13.5%) of analyzed patients and in 1/18 T-ALL cell lines (KARPAS-45) **(Table S6 and** **Figure 4** **)**. The somatic status of detected *TET1* variants was confirmed for 1 case (H1297Y) where a remission sample was available. We also investigated the variants in *TET2* and *TET3* picked up by 454 and performed additional Sanger sequencing for these genes. *TET2* variants were detected in 2 cell lines (JURKAT and KARPAS45) and one *TET3* variant was detected in the CCRF-CEM cell line, no T-ALL patient samples (0/46) harbored acquired TET2 or TET3 mutations **(Table S6)**.

**Figure 5:**
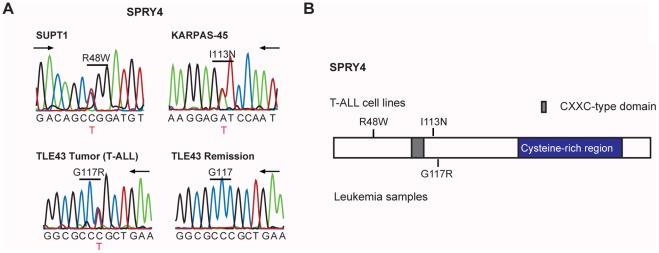
SPRY4 mutations. (A) Sanger sequencing chromatograms showing confirmed SPRY4 variants. (B) Domain structure of the SPRY4 protein with indication of novel detected variants.

Mutations in tyrosine phosphatase genes, that act as negative regulators of tyrosine signaling, were identified in many T-ALL cell lines and also in several T-ALL patients. Additional mutations in SPRY genes, negative regulators of the RAS/MAPK pathway, were also detected. We identified a homozygous variation in *SPRY3* in one T-ALL patient sample, and 3 mutations in *SPRY4* (2 mutations in cell lines and 1 somatically acquired mutation in a T-ALL patient sample). Sanger sequencing confirmed the presence of these mutations, but did not reveal any additional mutations of SPRY3/SPRY4 in 22 additional T-ALL cases, bringing the SPRY4 mutation frequency to 1/37 T-ALL patients and 2/18 T-ALL cell lines **(Table S7,** **Figure 5** **)**.

Finally, we also identified several mutations in tyrosine kinases (IGF1R, TYK2, TNK1, and MST1R) and associated signaling proteins (IRS2, SOCS3), but the majority of these mutations were found in cell lines, while primary patient samples showed a much lower frequency of these mutations. The most frequently mutated gene across all cell lines and patient samples was the insulin receptor substrate 2 (IRS2) gene, showing non-synonymous coding mutations in 6 cell lines and in one patient sample. Also frequently mutated was TYK2, with mutations observed in 6 cell lines; one stop-gain variant and 5 non-synonymous coding variants. Although none of the 15 patient samples carried a mutation in TYK2, it could be present at low frequency in patients. To test this, we performed complementary sequencing of TYK2 in 93 T-ALL, 54 AML and 53 B-ALL patient samples. Despite the high frequency of TYK2 variations in T-ALL cell lines, TYK2 variants were detected only in 2 of 93 T-ALL and 1 of 54 AML cases **(Table S5,** **Figure 3.C-D** **)**.

### Evidence for the Accumulation of Specific Mutations During in vitro Culture of the T-ALL Cell Lines

The mutation frequency of TYK2 in T-ALL cell lines compared to primary T-ALL samples was substantially different, with a high mutation rate of TYK2 in cell lines, but only a low mutation rate in primary samples. To determine if this could be due to the accumulation of TYK2 mutations during culturing of the cells, we sequenced TYK2 in different clones of the same T-ALL cell line **(** **Table 1** **)**. For the CCRF-CEM cell line, we obtained 5 different subclones that were collected over the years. Interestingly, whereas the R1027H variant was present in all analyzed samples, the A35V variant was only present in our line and in one additional CCRF-CEM clone. In the KARPAS-45 cell line, the Q830* variation was present in 3 different clones. In contrast, only our JURKAT line contained the C192Y mutation, while this was absent in 2 other clones available at DSMZ (www.dsmz.de) **(** **Table 1** **)**. These data suggest that at least some TYK2 mutations were acquired during extended cultivation of the cells, and thus are unlikely to represent an oncogenic event important for the development of leukemia *in vivo*. In addition, analysis of the transforming properties of these mutants in Ba/F3 cells could not identify major differences between wild type TYK2 and variants of TYK2 detected in cell lines or patient samples and we could not show any autophosphorylation of TYK2 in T-ALL cell lines containing TYK2 variants (data not shown).

These data confirm important differences between cell lines and primary patient samples, which may reflect the accumulation of mutations during *in vitro* cell culture.

**Table 1:** Analysis of TYK2 variants in cell lines over time and in different subclones.

Cell line	Tested variant	Result
CCRF-CEM Cools lab	R1027H	present
CCRF-CEM 2011 DSMZ (ACC240)	R1027H	present
CCRF-CEM subclone 1 DSMZ	R1027H	present
CCRF-CEM subclone 2 DSMZ	R1027H	present
CCRF-CEM subclone 3 DSMZ	R1027H	present
CCRF-CEM subclone 4 DSMZ	R1027H	present
CCRF-CEM subclone 5 DSMZ	R1027H	present
CCRF-CEM Cools lab	A35V	present
CCRF-CEM 2011 DSMZ (ACC 240)	A35V	present
CCRF-CEM subclone 1 DSMZ	A35V	absent
CCRF-CEM subclone 2 DSMZ	A35V	absent
CCRF-CEM subclone 3 DSMZ	A35V	absent
CCRF-CEM subclone 4 DSMZ	A35V	absent
CCRF-CEM subclone 5 DSMZ	A35V	absent
KARPAS-45 Cools lab	Q830*	present*
KARPAS-45 2011 DSMZ (ACC105)	Q830*	present*
KARPAS-45 1994 DSMZ (ACC105)	Q830*	present*
JURKAT Cools lab	C192Y	present
JURKAT 2011 DSMZ (ACC 282)	C192Y	absent
JURKAT 1992 DSMZ (ACC 282)	C192Y	absent

Presence of the TYK2 R1027 and A35V variants was tested in the CCRF-CEM cell line from our group (“CCRF-CEM Cools lab”) as well as in the CCRF-CEM cell line as it is currently sold by DSMZ (“CCRF-CEM 2011 DSMZ (ACC240)) and in 5 different CCRF-CEM subclones that DSMZ collected over the years. Similarly, KARPAS-45 from the Cools lab and the KARPAS-45 lines obtained from DSMZ in 2011 and in 1994 were screened for presence of the TYK2 Q830* variant. JURKAT cells from the Cools lab as well as JURKAT provided by DSMZ in 2011 and 1992 were tested for the TYK2 C192Y variant.

* This cell line has 4 copies of chromosome 19 containing TYK2. The height of the variant peak on the chromatogram suggests that only 1 copy of TYK2 contains the Q830* variant.

## Discussion

We demonstrated that the targeted sequencing approach with an optimized analysis setting can be used to identify oncogenic mutations. This approach could be of particular interest for the detection of point mutations in a set of important oncogenes and tumor suppressors or other disease related genes for diagnosis, prognosis prediction or therapy choice. Such information could be generated in a relatively short timeframe and with unprecedented detail. One of the major advantages over classical Sanger sequencing is the higher throughput of this method allowing that all exons of a gene set of this size can easily be sequenced. As such, full information is provided and rare variants or even previously undiscovered mutations in a particular gene can be detected. Indeed, of the 160 exonic and splice site variants (excluding the 61 synonymous variations) detected in the cell lines and patient samples across our panel of cancer genes, only 40 are found in the COSMIC database [16], of which 24 are associated specifically with T-ALL. Although for some genes mutation hotspots exist (e.g., the KRAS G12, G13, Q61 mutations), the function of most cancer genes can be affected by mutations at different positions. Therefore, for most cancer genes the entire coding sequence needs to be re-sequenced, and for this the Roche/454 technology is particularly suitable.

To detect mutations using next-generation sequencing - either to replace or complement molecular diagnosis - standardized bioinformatics analysis pipelines with very high accuracy are required. Such a pipeline consists of a mapping algorithm to align the sequence reads to the reference genome, a variation calling algorithm to identify differences between the sample and the reference, and a variation filtering algorithm.

We compared multiple combinations of mapping and variation calling algorithms, and found that combining two mappers, namely SSAHA-2 and BWA-SW, followed by Atlas-SNP2 yields the most accurate variation detection results. Adding two mapping algorithms filters out false positive variant predictions due to erronous mapping, and the error model of Atlas-SNP2 enables the elimination of reads that have multiple best matches in the reference genome. We also found that additional data filters on depth of coverage and on variant allele frequency further increased both the sensitivity and specifity of variation detection.

We encountered several technical limitations during data analysis. First, we had to remove duplicate reads introduced by PCR amplification steps during sample preparation since we noticed these were causing false positive SNV predicitons. Second, we could only predict SNVs, while indels (small insertions and deletions) had to be ignored since our work (data not shown) and previous studies indicate that 454 reads are not suited for indel detection due to the large amount of false positive results [4]. In a diagnostic setting, where 100% specificity is pursued, it is critical to identify genes or regions in genes that are prone to acquisition of indels and to design alternative assays to investigate them. Likewise, genomic rearrangements are important causes of T-ALL but require complementary detection technologies.

We believe that using a long read sequencing technology, such as Roche/454 or the more recent Pacific Bioscience, provides particular advantages with regards to both sensitivity and specificity of variation detection. First, long read alignment allows better distinction between highly similar genes in the genome. For example, one of the genes we re-sequenced was NOTCH1, a gene with multiple homologs (namely NOTCH2, NOTCH2Nl, NOTCH3 and NOTCH4). However, we observed no reads mapping to any of these homologs, even though we mapped the reads to the entire genome. This indicates that both the sequence capture and the mapping were specific. On the other hand, we also encountered an example where the sequence capture was not specific. Namely, the PMS2 gene is one of the targeted genes in our study, yet we observed reads mapping to the PMS2 pseudogene, PMS2CL, which contains the first six exons of PMS2 gene. Thanks to the use of long reads, this causes no problems for variation detection because for each gene the respective reads mapped *uniquely* to the correct gene, either PMS2 or PMS2CL. Note that the capture technology provides additional cues to achieve higher specificity because not only the exons are covered in the capture but also the flanking intronic regions. Therefore, the alignment is ‘aided’ by the intronic regions, where sequence similarity between homologs is lower, allowing for the reads to be correctly attributed to their origins in the genome.

Second, mapping long reads to a reference genome is more robust towards extensive local variation, which can be present in particular genomic regions, or can be higher when samples are sequenced from a different ethnicity compared to the reference genome [28]. We indeed found several regions that contain a high number of SNPs within a short sequence window. For example, there are 22 SNP clusters across all samples in a window of 200 bp with at least 3 SNPs, and 5 distinct clusters in a window of 100 bp with at least 3 SNPs. Figure S3 shows several examples, such as cluster of three SNPs within 100 bp in the SUMF1 gene in the ALLSIL cell line, and a cluster of 4 SNPs in a 200 bp window in the PTPRM gene in CCRF-CEM cell line. Nevertheless, in both cases a high coverage is obtained (36x and 46x respectively). These examples show that long reads enable a correct alignment and variation discovery, in contrast to short read sequencing technologies for which the mapping algorithms usually allow for a maximum of two mismathes per read.

We applied our analysis strategy to T-ALL by sequencing a set of 97 genes. This set consists of 58 known oncogenes and tumor suppressors in T-ALL and other cancers, and 39 genes selected via a candidate approach. Regarding the identification of variations in these genes using 454 sequencing and our optimal optimized analysis pipeline, we reached 95% sensitivity and 93% specificity on a confirmation set of 210 variants validated by capillary sequencing. Furthermore, we detected 85.7% of the mutations reported in 11 cell lines that were also sequenced in the Cancer Cell Line project. High performance of our resequencing approach is also illustrated by the fact that we identified mutations in known candidate drivers in T-ALL that were included in the collection of known cancer genes such as *NOTCH1* [29], *FBXW7* [30], *PTEN* [31], *PHF6* [14], *WT1* [32], [33] and *PIK3CA* [34].

We detected mutations in several known cancer genes where a link to T-ALL has not been established yet, such as JAK3. Interestingly, a recent article confirmed the mutation status of this gene in the context of T-ALL [20]. We also identified novel mutations in genes that were not previously associated with T-ALL tumorigenesis such as TET1, SPRY3 and SPRY4.

It is remarkable that more novel sequence variants are found per cell line sample than per patient, and that genes were in general more frequently mutated in cell lines than in patients. Excessive gene mutations can be explained by potential genomic instability of cells in culture, or can be caused by *in vitro* cell culture conditions. This hypothesis could be confirmed for *TYK2*, a very striking example for which 7/18 (38%) T-ALL cell lines contain novel *TYK2* sequence variants as opposed to only 2/93 (2%) T-ALL patients. Interestingly, we could demonstrate that several *TYK2* variants in cell lines had been acquired during culture. It remains to be determined what is promoting the frequent acquisition of *TYK2* variants in these T-ALL cell lines as opposed to T-ALL patients. The most obvious explanation are differences between the *in vitro* cell culture conditions and the physiological environment of T-ALL cells. As several cytokine signaling pathways depend on TYK2, presence of different cytokines and/or different concentrations of cytokines that use TYK2 signaling might be critical. These observations underscore once more that data obtained from cell culture models should be interpreted with care, especially when extrapolating these data to patient samples.

It is nevertheless interesting to note that this tendency of higher mutation frequency in cell lines compared to patient samples does not extend to all analyzed genes. The most evident example is *TET1*, showing novel variants in only 1/18 cell lines (KARPAS-45) versus 5/37 (13.5%) patients.

In conclusion, we describe a method for fast re-sequencing of a moderate size gene set of 97 genes using 454 next generation sequencing equipment that would be suitable for implementation into the clinic. Our results show that this setting is useful to identify (i) known mutations in known driver genes; (ii) new mutations in known drivers; and (iii) oncogenes or tumor suppressors that had not previously been associated with a specific subtype of cancer based on a candidate gene approach.

The optimized data analysis pipeline, which was assembled from publicly available tools, slightly exceeded the performance of the Roche gsMapper software with 95% sensitivity and 93% specificity for SNV detection, and subsequent analysis of the Roche/454 data from the T-ALL cell lines and patient samples confirmed previously known oncogenes and tumor suppressors in T-ALL and identified previously unrecognized rare somatic mutations in *TET1* and *SPRY4* in T-ALL patients. Screening a larger patient series should reveal the exact mutation frequency of these genes in T-ALL and whether mutations in these tumor suppressors also play a role in other types of hematopoietic malignancies.

## Materials and Methods

### Targeted Genes

97 genes were selected for sequencing in this study. The gene set consists of genes that are known to be involved in oncogenesis of T-ALL (and other cancer types), and a large set of kinases and phosphotases due to their potential therapeutic value. In total, 56 of the selected genes have been causally implicated in cancer according to Census [2] (extracted on 10th of November 2011) and 81 have somatic mutations in cancer according to COSMIC [16] (v48 release). According to the Molecular Signature Database [35] extracted on 10th of September 2010) there are 40 tumor suppressors and 32 oncogenes among the targeted 97 genes. Twenty of the 24 known cancer genes from the NCI-60 cell line set [36] are also included in the selected genes. Functional classification of the genes performed with DAVID [37] shows enrichment for GO terms related to cell proliferation and to signaling cascades, besides the expected enrichment for kinase and phosphotase activity **(Figure S4; Table S8**).

### Cell Lines and Patient Samples

All T-ALL cell lines originated from DSMZ (Braunschweig, Germany). Samples from patients with T-ALL (n = 93), Acute myeloid leukemia (AML) (n = 54) and B-cell acute lymphoblastic leukemia (B-ALL) (n = 53), obtained at diagnosis and remission samples from T-ALL patients (n = 42) were collected at the University Hospital Leuven and VU Medical Center Amsterdam. Diagnosis of T-ALL, AML or B-ALL was based on morphology, cytogenetics and immunophenotyping according to the World Health Organization and European Group for the Immunological Characterization of Leukemias (EGIL) criteria. Informed consent was obtained from all subjects and experiments were approved by the ethical committee of the University Hospital Leuven.

### Sequence Capture and Pyrosequencing

Preparation of a shot-gun DNA sequencing library and capture of the exons, with flanking intron junctions of 97 genes (**Table S9**) was performed on custom designed Nimblegen sequence capture 385K Version 2.0 Arrays (Roche Applied Science, Mannheim, Germany) according to the manufacturer’s instructions. The content of these arrays is described in (**Table S9**). Captured DNA was pyrosequenced on a GS FLX instrument (Roche).

### Evaluation of the Alignment and Variant Calling Algorithms

The performance of the alignment and variant calling algorithms was evaluated to determine the optimal method for analyzing 454 reads. Eight analysis pipelines were constructed from long read aligners BWA-SW [1], SSAHA2 [7], BLAT [8]-ERROR; and variant callers SAMTools [9], VarScan [10], Atlas-SNP2 [11]. (We will use the term ‘pipeline’ to refer to a combination of an aligner and a variant caller in the remaining part of this manuscript.) In addition to these pipelines, gsMapper was also included in the evaluation. The aligners map the sequence reads to the human reference sequence (NCBI Build 36.1). To remove duplicate reads in the data, the alignments generated by SSAHA2 and BWA-SW were processed further using Picard [38] (BLAT alignments were not “dedupped” since Picard requires the alignments in BAM format, and format conversion was not possible). Reads mapping to multiple locations in the reference genome (possibly coming from the homologes and/or pseudogenes of the genes targeted in the capture) are marked with a mapping quality of 0.

The pipelines were implemented and reviewed on 7 cell lines: *P12ichikawa*, *KE-37*, *ALL-SIL*, *CCRF-CEM*, *KARPAS45*, *SUPT1*, *DND41*.

Initial SNV predictions were performed with following settings:

- SAMTools: with pileup -c command, with total coverage threshold of 3 and SNP quality threshold of 20
- VarScan: with pileup2snp –min_coverage 3–min_reads2 2 min_avg_qual 15–min_var_freq 0.01–p_value 0.99
- Atlas-SNP2: with total coverage threshold of 3
- gsMapper: with *HCDiff* (high confidence differences) strategy; requiring the following criteria:
  1. There must be at least 3 reads with the difference.
  2. There must be both forward and reverse reads showing the difference, unless there are at least 5 reads with quality scores over 20 (or 30 if the difference involves a 5-mer or higher).
  3. If the difference is a single-base overcall or undercall, then the reads with the differences must form the consensus of the sequenced reads.

The SNPs to be confirmed with capillary sequencing were selected from the predictions generated with these settings.

Then, predictions from the pipelines were filtered with varying VAF and DoC thresholds. Two VAF thresholds (0.20 and 0.30) and two DoC thresholds (3 and 10) were used. SAMTools pipelines were also processed with *samtools varFilter* command, which implements minimum RMS mapping quality of 25, minimum read depth of 3, maximum read depth of 100, SNP within 10 bp around a gap to be filtered, and maximum number of SNPs in a window of 10 bp to be 2. Atlas-SNP2 pipelines were also filtered with posterior SNP-probability threshold P(SNP|Sj,cj).

The performance of each pipeline was evaluated by Sanger resequencing of 210 variants that were sampled from the pooled set of all predicted variants from all pipelines **(Table S10)** and the performance of each pipeline was quantified by calculating sensitivity, specificity and Matthews correlation coefficient (MCC) which ranges from 0, no correlation, to 1, perfect correlation [39].

### Sanger Sequencing

Whole genome amplified DNA (REPLI-g system, Qiagen, Hildenberg, Germany) from primary leukemia or remission samples was used as template for PCR amplification of indicated genes. PCR products were Sanger sequenced and inspected for the presence of sequence variants using Mutation Surveyer software (Softgenetics, State College, PA) and CLC DNA Workbench 6 (CLC Bio, Aarhus, Denmark). All variants that were detected in whole genome amplified material were subsequently validated in non-amplified original patient material. Primer sequences are available upon request.

### Data Availability

Sequence data has been deposited at the European Genome-phenome Archive (EGA, http://www.ebi.ac.uk/ega/), which is hosted by the EBI, under accession number EGAS00001000268.

## Supporting Information

Figure S1 **Mis-alignment in SSAHA2 causes a false prediction in TET2.** IGV (Integrative Genomic Viewer) software visualizes alignment of next generation sequencing reads to the reference sequence.15 In IGV, each sequence read is represented as a grey rectangle and the reference sequence is represented at the bottom. If there is base in a read that is different from the reference sequence, it is indicated with the corresponding letter. This figure shows the IGV output when anlayzing the same set of reads with the BWA-SW (top) and with the SSAHA2 (bottom) algorithms for sequence alignment. Looking at the alignments generated by these two algorithms revealed that SSAHA2 alignment was incorrectly positioning 3 reads (as indicated by colored read names on the plot), causing a false variant call on chr4:106384366 and resulting false prediction of a non-synonymous coding mutation in the TET2 gene.
(PDF)

Figure S2 **Low coverage exons have significantly higher GC-content.** Comparing the GC-content of the exons with low and high coverage revealed that the two groups have significantly different GC-content (p-value 2.2e-16), with low coverage exons having higher GC-content.
(PDF)

Figure S3 **SNP clusters identified in (A) SUMF1 gene in ALLSIL cell line and (B) PTPRM gene in CCRF-CEM cell line.**
(PDF)

Figure S4 **Functional classification of the 97 selected genes based on molecular function terms.**
(PDF)

Table S1 **Performance comparison of different analysis pipelines and parameter settings.**
(XLSX)

Table S2 **Performance of the pipelines under varying parameters.**
(XLSX)

Table S3 **368 retained SNVs.**
(XLSX)

Table S4 **Point mutations from the Cancer Cell Line project and their detection status by different analysis pipelines.**
(XLSX)

Table S5 **Sanger confirmed variants in JAK genes.**
(XLSX)

Table S6 **Sanger confirmed variants in TET genes.**
(XLSX)

Table S7 **Sanger confirmed mutations in SPRY genes.**
(XLSX)

Table S8 **Top 20 enriched (a) molecular function and (b) biological process GO terms from 97 genes.**
(XLSX)

Table S9 **97 selected genes for capture and their targeted exons.**
(XLSX)

Table S10 **Selected positions for Sanger sequencing validation.**
(XLS)
