# Supplementary material for: High Accuracy Mutation Detection in Leukemia on a Selected Panel of Cancer Genes
Source: PLoS One. 2012 Jun 4;7(6):e38463. doi: 10.1371/journal.pone.0038463 (PMC3366948; doi:10.1371/journal.pone.0038463)

Figure S1: Mis-alignment in SSAHA2 causes a false prediction in *TET2*.

IGV (Integrative Genomic Viewer) software visualizes alignment of next generation sequencing reads to the reference sequence.[15] In IGV, each sequence read is represented as a grey rectangle and the reference sequence is represented at the bottom. If there is base in a read that is different from the reference sequence, it is indicated with the corresponding letter. This figure shows the IGV output when anlayzing the same set of reads with the BWA-SW (top) and with the SSAHA2 (bottom) algorithms for sequence alignment. Looking at the alignments generated by these two algorithms revealed that SSAHA2 alignment was incorrectly positioning 3 reads (as indicated by colored read names on the plot), causing a false variant call on chr4:106384366 and resulting false prediction of a non-synonymous coding mutation in the *TET2* gene.

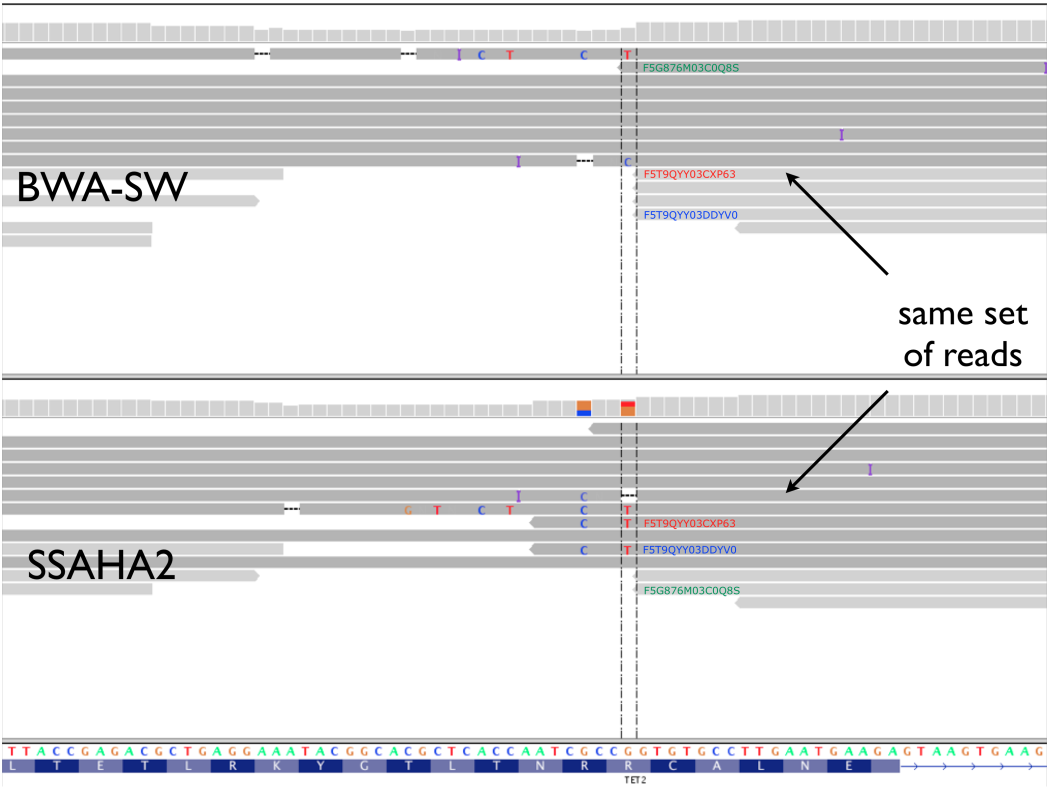

Supplement: Figure S1 — Mis-alignment in SSAHA2 causes a false prediction in TET2. IGV (Integrative Genomic Viewer) software visualizes alignment of next generation sequencing reads to the reference sequence.15 In IGV, each sequence read is represented as a grey rectangle and the reference sequence is represented at the bottom. If there is base in a read that is different from the reference sequence, it is indicated with the corresponding letter. This figure shows the IGV output when anlayzing the same set of reads with the BWA-SW (top) and with the SSAHA2 (bottom) algorithms for sequence alignment. Looking at the alignments generated by these two algorithms revealed that SSAHA2 alignment was incorrectly positioning 3 reads (as indicated by colored read names on the plot), causing a false variant call on chr4:106384366 and resulting false prediction of a non-synonymous coding mutation in the TET2 gene. (PDF) [file pone.0038463.s001.pdf]
