# Supplementary material for: High Accuracy Mutation Detection in Leukemia on a Selected Panel of Cancer Genes
Source: PLoS One. 2012 Jun 4;7(6):e38463. doi: 10.1371/journal.pone.0038463 (PMC3366948; doi:10.1371/journal.pone.0038463)

Figure S2: Low coverage exons have significantly higher GC-content.

Comparing the GC-content of the exons with low and high coverage revealed that the two groups have significantly different GC-content (p-value 2.2e-16), with low coverage exons having higher GC-content.

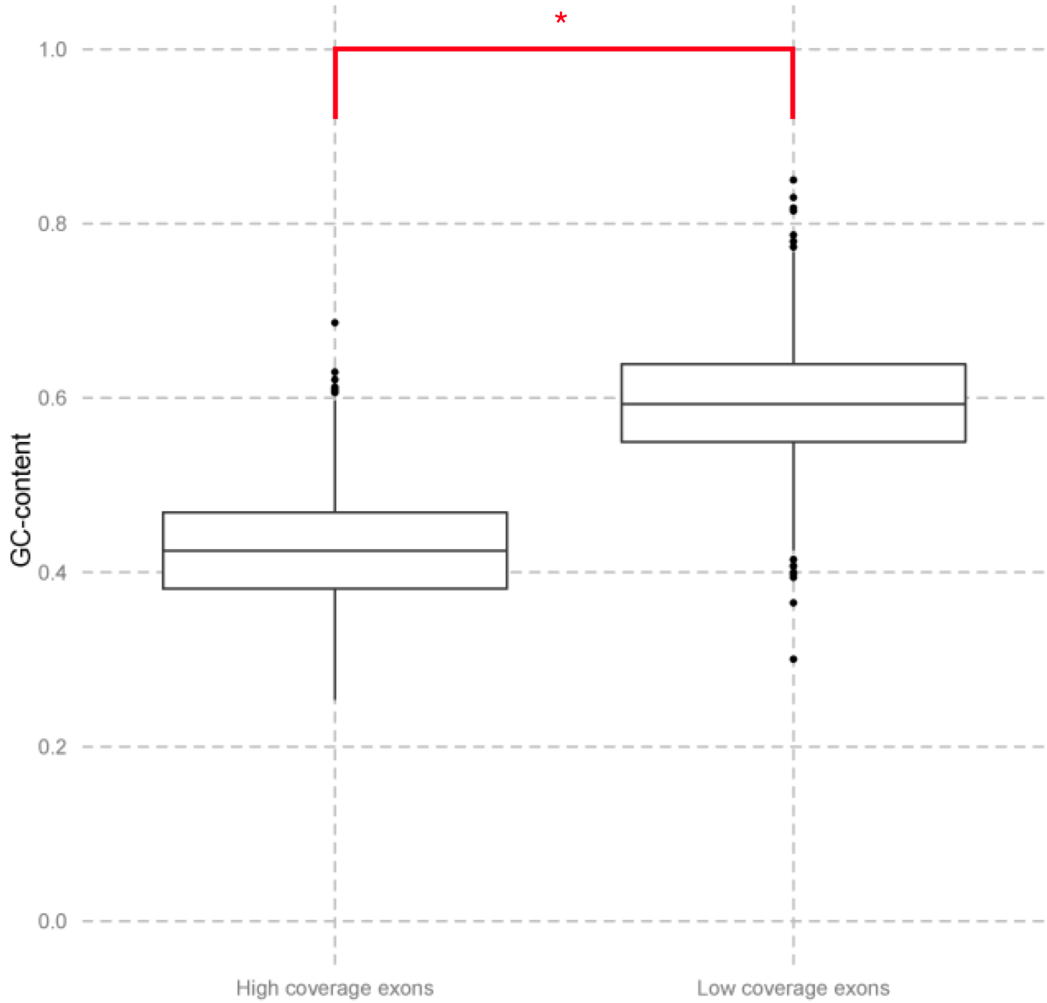

Supplement: Figure S2 — Low coverage exons have significantly higher GC-content. Comparing the GC-content of the exons with low and high coverage revealed that the two groups have significantly different GC-content (p-value 2.2e-16), with low coverage exons having higher GC-content. (PDF) [file pone.0038463.s002.pdf]
