# Supplementary material for: High Accuracy Mutation Detection in Leukemia on a Selected Panel of Cancer Genes
Source: PLoS One. 2012 Jun 4;7(6):e38463. doi: 10.1371/journal.pone.0038463 (PMC3366948; doi:10.1371/journal.pone.0038463)

Figure S3: SNP clusters identified in (A) SUMF1 gene in ALLSIL cell line and (B) PTPRM gene in CCRF-CEM cell line

(A)

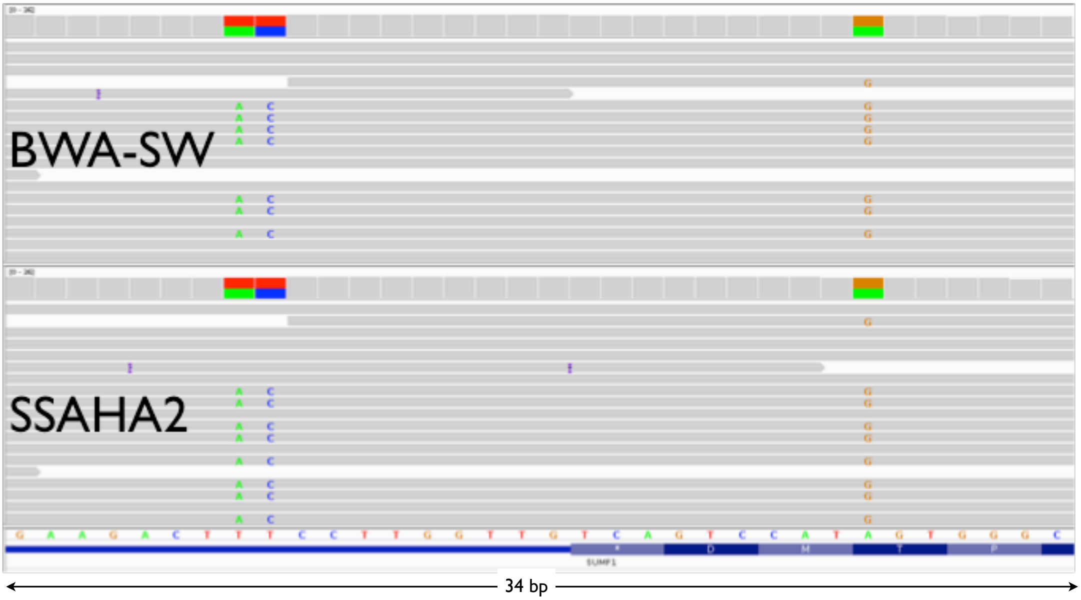

(B)

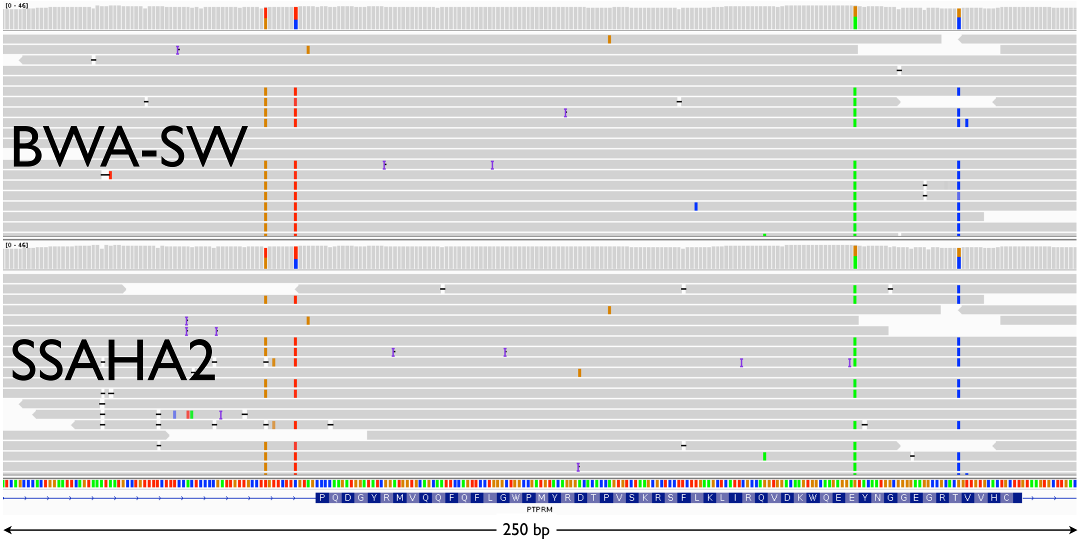

Supplement: Figure S3 — SNP clusters identified in (A) SUMF1 gene in ALLSIL cell line and (B) PTPRM gene in CCRF-CEM cell line. (PDF) [file pone.0038463.s003.pdf]
