# Supplementary figures and images for: High Accuracy Mutation Detection in Leukemia on a Selected Panel of Cancer Genes
Source: PLoS One. 2012 Jun 4;7(6):e38463. doi: 10.1371/journal.pone.0038463 (PMC3366948; doi:10.1371/journal.pone.0038463)

**Figure S4:** Functional classification of the 97 selected genes based on molecular function terms.

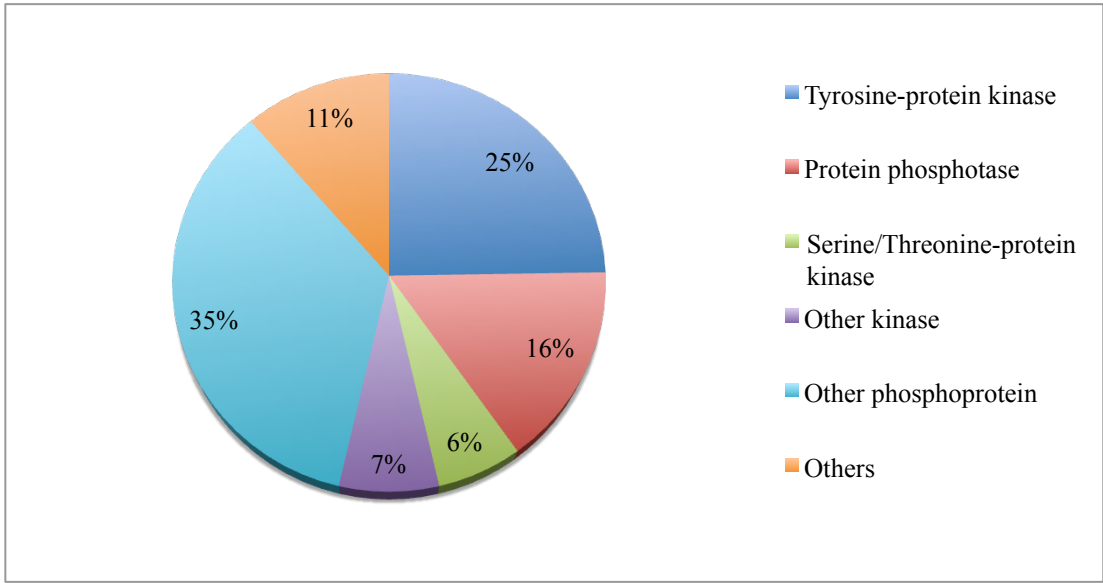

Supplement: Figure S4 — Functional classification of the 97 selected genes based on molecular function terms. (PDF) [file pone.0038463.s004.pdf]
